# Statistical Analysis of Methotrexate Degradation by UV-C Photolysis and UV-C/TiO_2_ Photocatalysis

**DOI:** 10.3390/ijms24119595

**Published:** 2023-05-31

**Authors:** Luis A. González-Burciaga, Juan C. García-Prieto, Cynthia M. Núñez-Núñez, José B. Proal-Nájera

**Affiliations:** 1 CIIDIR-Unidad Durango, Instituto Politécnico Nacional, Calle Sigma 119, Fracc. 20 de Noviembre II, Durango 34220, Mexico; 2 Centro de Investigación y Desarrollo Tecnológico del Agua, Universidad de Salamanca, Campo Charro s/n, 37080 Salamanca, Spain; 3 Ingeniería en Tecnología Ambiental, Universidad Politécnica de Durango, Carretera Durango-México km 9.5, Col. Dolores Hidalgo, Durango 34300, Mexico

**Keywords:** methotrexate, heterogeneous photocatalysis, cytostatic, water treatment, photocatalyst

## Abstract

Methotrexate (MTX) is a folic acid analog and has been used to treat a wide variety of malignant and non-malignant diseases. The wide use of these substances has led to the continuous discharge of the parent compound and its metabolites in wastewater. In conventional wastewater treatment plants, the removal or degradation of drugs is not complete. In order to study the MTX degradation by photolysis and photocatalysis processes, two reactors were used with TiO_2_ as a catalyst and UV-C lamps as a radiation source. H_2_O_2_ addition was also studied (absence and 3 mM/L), and different initial pHs (3.5, 7, and 9.5) were tested to define the best degradation parameters. Results were analyzed by means of ANOVA and the Tukey test. Results show that photolysis in acidic conditions with 3 mM of H_2_O_2_ added is the best condition for MTX degradation in these reactors, with a kinetic constant of 0.028 min^−1^. According to the ANOVA test, all considered factors (process, pH, H_2_O_2_ addition, and experimentation time) caused statistically significant differences in the MTX degradation results.

## 1. Introduction

The Environmental Protection Agency (EPA) defines emerging pollutants (EPs) as new types of chemical compounds that are not regulated and whose impact is not fully understood [1,2]. These persistent chemical compounds are not monitored in the environment, but they can produce adverse health effects [3]. Due to the above, the NORMAN database was created to monitor and bio-monitor emerging substances in the environment, aimed at laboratories, research centers, and organizations specialized in monitoring EPs [4]. The NORMAN database has identified more than 700 compounds that have been classified into various groups such as drugs, personal care products, disinfectants, detergents, pesticides, hormones, and drugs, among others [2,3,4].

One of the groups of EPs that has gained great importance are drugs that are widely used in medicine (human and veterinary) and aquaculture. The wide use of these substances has led to the continuous discharge of the parent compound and its metabolites in wastewater [5]. In conventional wastewater treatment plants (PTAR), the removal or degradation of drugs is not complete, and concentrations of 0.008 to 55.78 μg/L have been detected. The accumulation of these compounds can cause reproductive problems and the inhibition of cell division in aquatic or terrestrial organisms that come into contact with them [6].

Drugs for the treatment of cancer, also called cytostatic drugs (CD), represent a worrying health risk due to the increase in their demand due to the increase in cases of patients with this condition. Its carcinogenic, genotoxic, mutagenic, and cytotoxic characteristics at low concentrations, together with its low biodegradability, have aroused the interest of researchers to monitor its presence in the environment and its toxic potential [7,8,9,10].

Methotrexate (MTX) is a folic acid analog [11] and has been used to treat a wide variety of malignant and non-malignant diseases [12]. MTX was originally produced as an anticancer drug, and its ability to prevent cell proliferation has been tested in various types of cancer, such as leukemia, osteosarcoma, lymphoma, breast, and bladder cancer, and has even been tested in the treatment of some brain tumors [11,12,13,14]. The initial doses for the treatment of various types of cancer in adults administered in Mexico are 50 mg/m^2^ of body surface by the intravenous or intramuscular route and from 5 to 10 mg/m^2^ of body surface by the intrathecal route [15,16]. In some European countries, the United Kingdom, and the United States of America, doses can range from 20 to 40 mg/m^2^/day administered orally [17]. In addition to its chemotherapeutic use, MTX has anti-inflammatory properties, which is why it is used in the treatment of skin and joint disorders, especially moderate psoriasis and rheumatoid arthritis [18,19], where the doses used to treat these conditions are 30 mg/week and 20 mg/week, respectively [20,21].

It is well known that MTX is not completely metabolized once it is consumed, and up to 90% of the parent compound can be excreted through feces and urine after 24 h. Being a compound resistant to biodegradation, it can enter the water cycle through domestic and hospital wastewater, and its presence in drinking water has even been reported [8,9,22]. Due to its characteristics and its wide use in different conditions in high doses, the presence and degradation of the cytostatic drug MTX present in residual water are extremely important.

The most promising and popular technique for degrading recalcitrant contaminants of pharmaceutical origin is heterogeneous photocatalysis (HPC), due to its ability to produce hydroxyl radicals (∙OH) from the photocatalyst involved in the process. ∙OH are highly oxidizing agents (E° = 2.8 eV) that can carry contaminants to mineralization, producing harmless compounds such as CO_2_ and H_2_O [23,24,25,26]. The photocatalyst is the central part of the photocatalytic process, and most are metallic oxides such as TiO_2_, which is the most commonly used in environmental applications because it is biologically inert and resistant to chemical corrosion; it is also low cost, has a good ability to absorb light, and can be used at room temperature and pressure [27,28].

HPC can be defined as a series of simultaneous redox reactions on the surface of the photocatalyst. When irradiated with UV light with an energy equal to or greater than the bandgap, valence band electrons pass to the conduction band ($e_{\mathrm{CB}}^{-}$) leaving an empty area with a positive charge known as a hole ($h_{\mathrm{VB}}^{+}$). While $e_{\mathrm{CB}}^{-}$ reduces dissolved oxygen in the solution to produce ∙OH, $h_{\mathrm{VB}}^{+}$ is positive enough to convert water adsorbed on the photocatalyst to generate ∙OH [23,24,25,26].

The degradation of MTX by advanced oxidation processes has been studied in the past, but there is still a need to find an effective, innovative, and cheap method to degrade methotrexate. The objective of this work was to study MTX degradation through the application of photolysis and heterogeneous photocatalysis processes using UV-C lamps as radiation sources under different operating conditions (acid, neutral, and basic pH). The effect of adding hydrogen peroxide (H_2_O_2_) to processes as an oxidizing agent was also studied in order to determine the statistically significant factors that influence the process results.

## 2. Results and Discussion

Methotrexate is a folic acid analog and has been continually discharged into the environment as it is not considered in common treatments employed by wastewater treatment plants. Results here presented show that photolysis and photocatalysis can be efficiently employed to degrade MTX present in wastewater.

### 2.1. MTX Photolityc Degradation

Control experiments were performed in the absence of radiation, showing a maximum degradation of less than 1% after 120 min of reaction; therefore, the presence of H_2_O_2_ is not sufficient to degrade MTX if UV light is not involved in the process.

In the past, Lai et al. [10] (2017) stated that UV radiation does not break the MTX molecule, coinciding with our results, where photolysis alone showed very low degradation.

**Figure 1:**
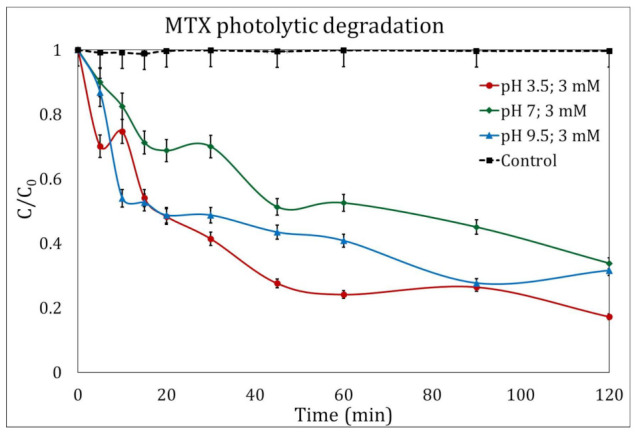
Photolytic degradation of methotrexate (MTX) in the presence of H_2_O_2_ (3 mM/L) at different pH magnitudes: 3.5 (circles), 7 (diamonds), and 9.5 (triangles). The control experiment is represented by the dotted line.

The degradation of MTX after two hours of UV radiation in the absence of H_2_O_2_ was less than 17% in the three magnitudes of pH evaluated. Figure 1 only shows the experiments to degrade MTX in the presence of H_2_O_2_ at a concentration of 3 mM/L, where the best results were obtained at a pH of 3.5, reaching a total degradation of 82.64%.

Direct photolysis of molecules is possible without the addition of any reagents, as a 254 nm photon possesses 4.89 eV of energy, enough to produce homolytic or heterolytic breakages of the molecules [29]. Nevertheless, such a reaction is too slow in this case to compete with a UV/H_2_O_2_ process.

Kinetic analysis showed kinetic constants of 0.028 min^−1^, 0.013 min^−1^ and 0.027 min^−1^ for the photolysis process when adding 3 mM of peroxide for experiments with initial pH of 3.5, 7 and 9.5, respectively. Calculated kinetic constants for photolytic MTX degradation without H_2_O_2_ addition was always lower.

Somensi et al. calculated rate constants for MTX degradation through ozonolysis of 0.3373 min^−1^ and 0.4163 min^−1^ through the sonolysis/ozonolysis process, both performed under pH 7 [30].

### 2.2. MTX Photocatalytic Degradation

Control experiments carried out with the presence of TiO_2_ and H_2_O_2_ in the absence of UV radiation showed a maximum degradation of 1.5% after 120 min of reaction, demonstrating that the presence of the photocatalyst is not enough to degrade MTX. Moreover, MTX does not absorb on the photocatalysts, as stated by Lai et al. [10].

**Figure 2:**
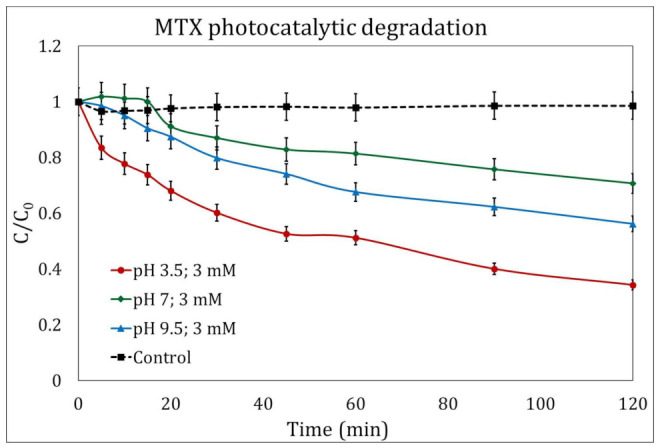
Photocatalytic degradation of methotrexate (MTX) in the presence of H_2_O_2_ (3 mM/L) at different pH magnitudes: 3.5 (circles), 7 (diamonds), and 9.5 (triangles). The control experiment is represented by the dotted line.

The photocatalysis experiments showed a similar behavior to the photolysis experiments (Section 2.1), and the addition of H_2_O_2_ (3 mM/L) greatly improved the efficiency of pollutant degradation. After 120 min of reaction, a maximum of 65.73% was reached at a pH of 3.5 (Figure 2). Similar results were obtained by Espinosa et al. [31], who achieved a 73% degradation of MTX at a pH of 3 and radiation with a wavelength of 254 nm.

Remarkably, photocatalytic kinetic constants were lower than those calculated for the photolytic process, with values for experiments when H_2_O_2_ was added of 0.016 min^−1^, 0.005 min^−1^, and 0.008 min^−1^ for initial pH values of 3.5, 7, and 9.5, respectively. 

In the past, Alinejad et al. [32] used ozonation processes and catalytic ozonation processes with a nitrate magnesium oxide nano-catalyst to degrade MTX; in such research, acidic and alkaline pH showed better degradation when compared to neutral initial pH. The best results were obtained at a pH near 8, reaching 87% degradation.

### 2.3. Statistic Analysis

From the degradation data, statistical analysis was performed to find the factors that influence the statistical significance of the degradation response variable. 

**Figure 3:**
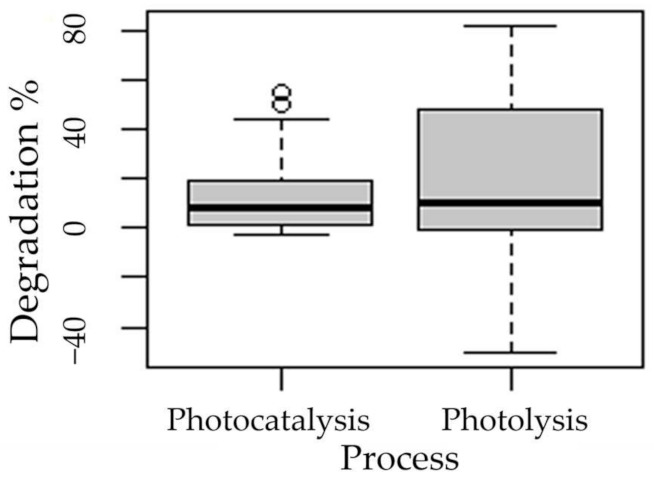
Boxplot of the degradation of methotrexate with respect to the process factor.

**Figure 4:**
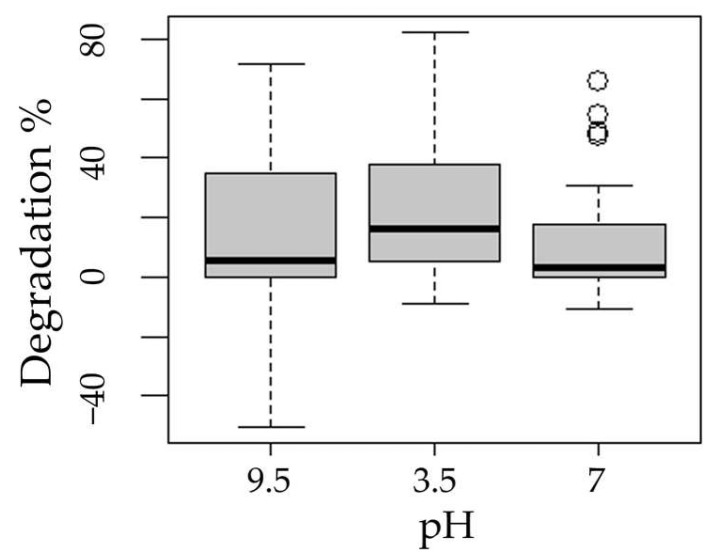
Boxplot of methotrexate degradation with respect to pH factor.

**Figure 5:**
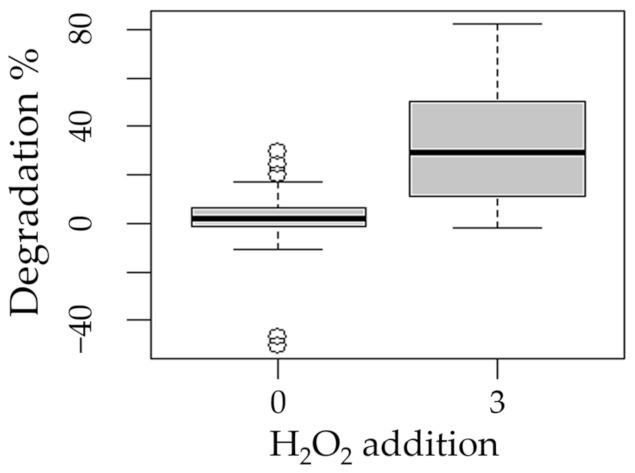
Boxplot of the degradation of methotrexate with respect to the factor addition of hydrogen peroxide (0 and 3 mM/L).

**Figure 6:**
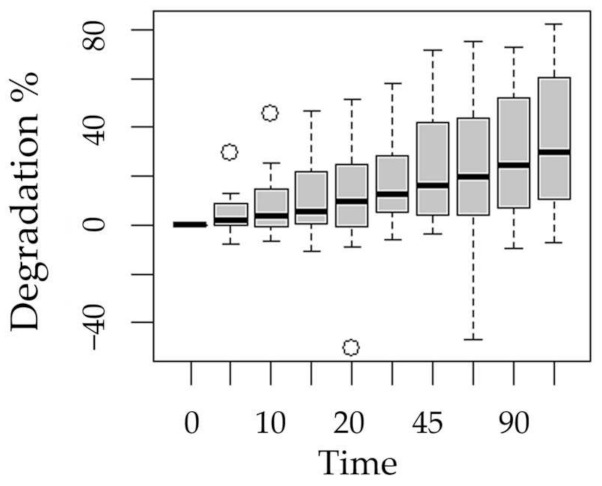
Boxplot of methotrexate degradation with respect to the experimentation time factor.

The following boxplots show the means of degradation with respect to the factors process (Figure 3), pH (Figure 4), presence of hydrogen peroxide (Figure 5), and experimentation time (Figure 6).

Direct irradiation of pollutant-containing water leads to the promotion of a pollutant molecule from the fundamental state to an excited singlet state, which may then intersystem cross to produce triplets. Such excited states can undergo homolysis, heterolysis, or photoionization, among other processes. In most cases, homolytic rupture produces radicals (Equation (1)):

(1) $$R-R+\mathrm{hv}\to R-R^{*}\to 2R^{\cdot }$$

These radicals initiate chain reactions to produce the final, low-weight products; in the presence of oxygen, additional reactions generating the superoxide radical are possible [29].

In the past, Kanjal et al. [33] found MTX photolysis degradation as low as 9% after 30 min of experimentation; in this research, 17% degradation under acidic conditions was achieved.

One of the disadvantages of photocatalysis is the high probability of recombination of the electron hole generated on the surface of the catalyst. Besides, as there is no physical separation between the anodic reaction site (oxidation by holes) and the cathodic one (reduction by electrons), back reactions can be of importance. Low efficiency is one of the most severe limitations of heterogeneous photocatalysis [29]. To avoid such recombination and improve HO∙ generation in situ, oxidative agents, such as H_2_O_2_, are added to the reaction [34]. 

In photolytic processes (UV and UV/H_2_O_2_), two degradation pathways are possible: direct photolytic breakage of pollutant and chain reactions started by the breakage of H_2_O_2_ in order to form HO∙ as portrayed in Equation (1) (when H_2_O_2_ was added). So, both possible degradation pathways compete for available UV radiation.

In photocatalysis (UV/TiO_2_ and UV/TiO_2_/H_2_O_2_), besides the pathways presented in photolysis, radiation excites the photocatalyst to form the electron hole (Equation (2)):

(2) $${\mathrm{TiO}}_{2}+\mathrm{hv}\to e^{-}+h^{+}$$

This fact could explain the results obtained in this study, where calculated reaction constants were higher for photolysis than those for photocatalysis (Section 2.2).

MTX has an extremely low solubility in water [35], is a weak dicarboxylic acid with a molecular weight of 454.5 g/mol and pKa values of 4.7 and 5.5 [36], and thus the isoelectric point for MTX was calculated to be 5.1. Compared with the 6.5 isoelectric point for TiO_2_, it is clear that the solution’s initial pH yields different results when studied. According to Babyszko et al. [37], the TiO_2_ surface remains positive under acidic pH, so it was expected that electrostatic repulsion, with both MTX and TiO_2_ positively charged, would decrease degradation given that heterogeneous photocatalysis is a surface phenomenon, but this was not the case.

The Tukey test applied to the pH factor showed significant differences between the low and high, neutral and high levels, but not in the neutral and low combination. Different effects of solution pH were expected, as, according to Alinejad et al. [32], solution pH is an important parameter given that most semiconductor oxides present amphoteric behavior.

Reguzzoni reported in 2017 [38] that the degradation of MTX with TiO_2_-P25/UV is not sensitive to pH, even when considering the zero charge point of the photocatalyst, which should favor attraction at low pH. In his research, it was established that the acidic pH hinders the degradation due to the coverage of the MTX on the TiO_2_ surface, inhibiting the photoactivation of the catalyst. In the present study, acidic pH actually improved photodegradation, so no evidence of such coverage was found.

According to Litter [29], the photochemical process is more efficient in alkaline media because the concentration of the conjugate anion of hydrogen peroxide increases with pH, and this species has a higher absorption coefficient than H_2_O_2_, favoring light absorption and increasing HO∙ production. In this study, slightly better results were demonstrated by acidic media.

When HNO_3_ (added in order to lower the pH of the solution for experiments with an acidic initial pH) dissociates in water, it yields ${\mathrm{NO}}_{3}^{-}$ and ${\mathrm{NO}}_{2}^{-}$, which, when excited under radiation, result in the formation of HO∙ [39]. Such a hydroxyl radical formation pathway could explain the better results obtained under acidic pH by posing an advantage over processes where no HNO_3_ was added.

When irradiated with short UV radiation, H_2_O_2_ can form hydroxyl radicals, according to the following equation (Equation (3)) [29,34,40,41]:

(3) $$H_{2}O_{2}\to 2\mathrm{HO}\cdot$$

So the additional path for hydroxyl radical formation was an important part of the experiments where H_2_O_2_ was added. 

In UV/H_2_O_2_ or UV/TiO_2_/H_2_O_2_ processes, hydroxyl radicals produced by hydrogen peroxide photolysis react with organic pollutants present. When the pollutants are acidic compounds, they may exist in a protonated form (R_1_H), and in addition to oxidation by hydroxyl radicals, they might be subject to direct photolysis under UV radiation (Equations (4)–(6)) [41]:

(4) $$\mathrm{HO}\cdot +{\;R}_{1}\to \mathrm{subproduct}$$

(5) $$\mathrm{HO}\cdot +{\;R}_{1}H\to \mathrm{subproduct}$$

(6) $$R_{1}+\mathrm{hv}\to \mathrm{subproduct}$$

Normally, H_2_O_2_ addition improves pollutant degradation, but when concentration is too high, it competes with pollutants by forming hydroxyl radicals, slowing down degradation by the formation of less reactive hydroperoxy radicals (Equation (7)) [29,41]:

(7) $$H_{2}O_{2}+\mathrm{HO}\cdot \to H_{2}O+{\mathrm{HO}}_{2}^{\cdot }$$

The statistical analysis showed that all factors considered (process, initial pH, peroxide addition, and experimentation time) were significant for the MXT degradation (*p* < 0.05) (Table 1).

### 2.4. Comparison of MTX Degradation, Residual H_2_O_2_ and TOC

The residual hydrogen peroxide in the photolysis experiments was 57.5%, while in the photocatalysis processes, the H_2_O_2_ consumption rose to 76%. 

**Figure 7:**
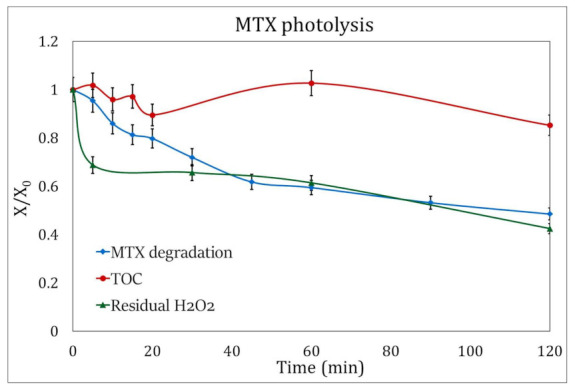
H_2_O_2_ consumption (green triangles) and evolution of TOC (red circles) during the degradation of MTX by photolysis (blue squares). The analysis was done under the best experimental conditions: pH 3.5 and addition of 3 mM H_2_O_2_.

**Figure 8:**
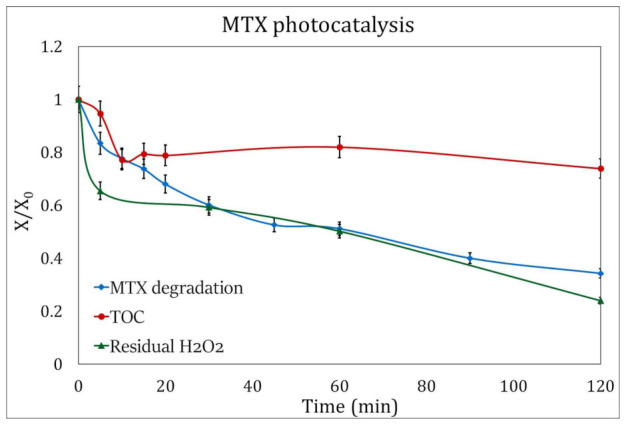
H_2_O_2_ consumption (green triangles) and TOC evolution (red circles) during MTX degradation by photocatalysis (blue squares). The analysis was performed under the best experimental conditions: pH 3.5 and addition of 3 mM H_2_O_2_.

Figure 7 and Figure 8 show the decrease in the concentration of the oxidizing agent during the duration of the experiments, and it can be observed that the initial concentration of H_2_O_2_ for these processes, at the experimental conditions presented here and 120 min of UV radiation, can be reduced to 2.3 mM/L for photocatalysis and 1.8 mM/L for photolysis.

The UV lamps used in this study, with an emission peak of 254 nm, do not represent an effective procedure for the total mineralization of pollutants [39], so low TOC removal was to be expected for the experiments.

### 2.5. MTX Degradation by Products

The analysis carried out by means of HPLC-MS of the samples obtained from the degradation of MTX by photolysis and photocatalysis at a pH of 3.5 and a concentration of 3 mM/L of H_2_O_2_ showed the existence of four possible degradation by-products.

There are three main mechanisms involved when a pollutant is attacked by a hydroxyl radical: hydrogen abstraction (generally the first step for acidic pollutants), OH addition or substitution, and electron transfer (Equations (8) and (9)) [29]:

(8) $$\mathrm{RH}+\mathrm{HO}\cdot \;\to H_{2}O+R\cdot$$

(9) $$R\cdot +O_{2}\to \mathrm{ROO}\cdot$$

If the target is an aromatic pollutant, the first step is ring hydroxylation, but further HO∙ reactions lead to the opening of the ring and the formation of open conjugated structures [29].

The formation of each by-product from MTX could follow different pathways: a ketonization at the carbon of position 11 produces by-product 1 with a higher molecular weight than the original compound (470.44 g/mol for by-product 1 versus 454.44 for the original MTX molecule). The cleavage at the nitrogen at position 21 removes the carboxylic groups from the molecule, forming by-product 2. The formation of a third and fourth by-product as a result of the cleavage at the nitrogen at position 12.

## 3. Materials and Methods

### 3.1. Sample Preparation and Reagents

The samples were produced by dissolving the drug MTX (Trxilem^®^, Lemery S.A. de C.V., Tlalpan, Mexico City, Mexico) in distilled water up to the desired concentration. For the photolysis experiments, 50 L samples were prepared, while for photocatalysis, the samples had a volume of 25 L, both with a 5 mg/L initial concentration of MTX. H_2_O_2_ was obtained from Labbox Labware (CAS: 7722-84-1, Barcelona, Catalonia, Spain). Titanium oxysulfate was purchased from Sigma-Aldrich (CAS: 13825-74-6, St. Louis, MO, USA). The MTX reagent for the calibration curves was purchased from Sigma-Aldrich (CAS: 59-05-2, St. Louis, MO, USA). 

### 3.2. MTX Degradation via Photolysis

**Figure 9:**
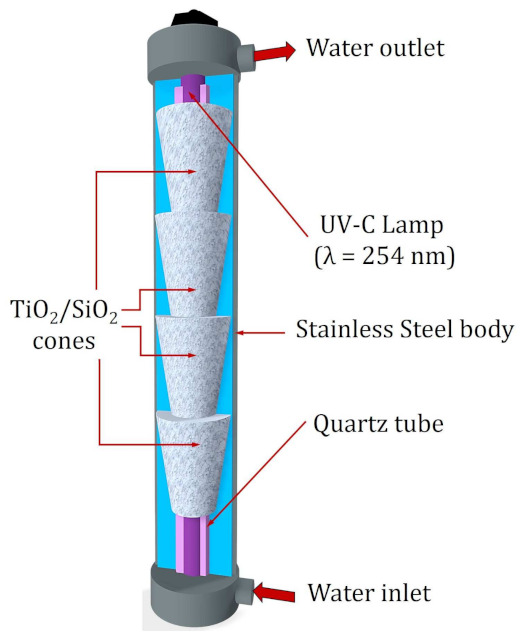
Components of the reactor used in the degradation of MTX by photolysis. Interior stainless-steel cones with the TiO_2_/SiO_2_ mesh were removed for photolysis experiments.

The system where the reactor is located consists of a 200 L tank, a pump with a power of 1 hp that allows recirculating the sample through the entire system, and a 50 μm filter. The main part of the system is a stainless-steel compartment that has a low-pressure mercury lamp (radiation peak of 254 nm, T5 Philips, Amsterdam, The Netherlands) as a source of UV radiation. A quartz tube surrounds the UV lamp to prevent it from coming into direct contact with the sample. Both components are in the center of the reactor, and in this way, the radiation is reflected by the polished inner surface of the stainless-steel body of the reactor to the photocatalyst and the sample. For photocatalysis experiments, the reactor has four stainless steel cones that contain a SiO_2_ mesh where the TiO_2_ is impregnated. For photolysis processes, as in this study, the cones are removed to avoid the presence of the photocatalyst (Figure 9) [42].

The experiments were carried out at a constant flow rate of 650 L/h. Once the recirculation through the system began, the pH was adjusted with a 65% HNO_3_ solution or a 0.1 M NaOH solution to obtain the desired magnitudes. (pH: 3.5, 7, and 9.5), which were selected considering the isoelectric point of TiO_2_ (6.5). 

The effect of H_2_O_2_ on the degradation of MTX was also analyzed; for this objective, the experiments were carried out either adding 3 mM/L of H_2_O_2_ or without H_2_O_2_ addition at each magnitude of pH. 

After adjusting the conditions in each experimental run (pH and H_2_O_2_ dose added when required), the initial sample (0 min) was taken and the lamp was turned on. During the experiments, sampling had the following time distribution: 5, 10, 15, 20, 30, 45, 60, 90, and 120 min, from which the analyses and quantification of MTX degradation were performed.

### 3.3. MTX Degradation via Photocatalysis

The photocatalysis experiments were carried out, testing the same factors as the photolytic processes: pH levels (3.5, 7, and 9.5) and H_2_O_2_ doses (absence and 3 mM/L). Samples were taken at the same experimentation times as established for photolysis. The flow rate was set to 500 L/h for photocatalysis experiments.

**Figure 10:**
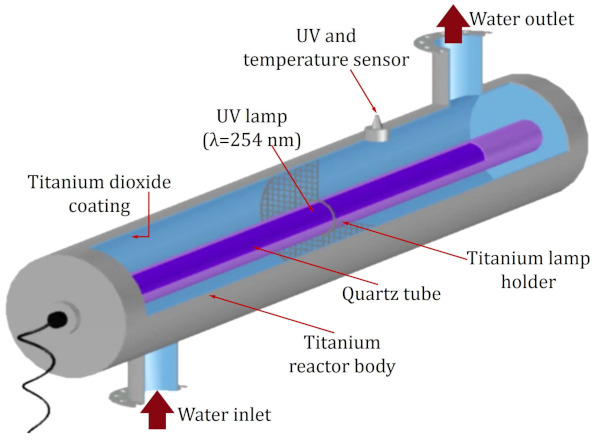
Internal structure of the AOP1 model reactor, used for the MTX degradation experiments by means of photocatalysis.

The reactor used for photocatalysis was the commercial model AOP1 (Bright-Water Environmental, Harleston, Norfolk, UK). Figure 10 shows the reactor, made up of a titanium cylinder with dimensions of 75 mm in diameter and 475 mm in length, covered on the inside by a layer of TiO_2_. A 254 nm radiation-emitting lamp was used as a radiation source.

### 3.4. Control Experiments

In order to evaluate the performance of the photolysis and photocatalysis processes in the degradation of MTX, the effect of H_2_O_2_ and the TiO_2_/H_2_O_2_ interaction on the contaminant must be ruled out; therefore, control experiments were carried out in the absence of radiation. The solutions were treated as described in Section 3.2 and Section 3.3, adding 3 mM/L H_2_O_2_, and the pH was adjusted to 6.5, the same as the TiO_2_ isoelectric point, also avoiding charge interaction. In these experiments, the lamp was not turned on.

### 3.5. Design of Experiments and Statistical Analysis

The MTX degradation was investigated as a function of four factors: process, initial pH, and H_2_O_2_ addition. Besides, experimentation time was considered as samples were taken at different moments during each experiment (0, 5, 10, 15, 20, 30, 45, 60, 90, or 120 min). The experimental design then consists of a 2 × 3 × 3 factorial design, with sampling at different times and at least 2 duplicates (Table 2).

The statistical analysis of the degradation data was performed using the R Studio language. To analyze the data, a multiple ANOVA was used, considering MTX degradation as the response variable and, as factors, the process, the initial pH, the addition of peroxide, and the experimentation time in which the sample was taken (Table 2). In addition, a Tukey test was performed to identify the levels of each factor with significantly different means.

### 3.6. MTX, Residual H_2_O_2_ and TOC Concentration Analysis

Samples were taken directly at the outlet of the solution tank just in connection with the rest of the system and analyzed by UV/vis spectrophotometry (T80+ UV/vis spectrophotometer, PG Instruments Ltd., Alma Park, Leicestershire, UK) at a wavelength of 303 nm to follow the degradation of MTX. The calibration curve was made with the MTX reagent level diluted in deionized water, and an R^2^ of 0.9996 was obtained.

The consumption of H_2_O_2_ during the experiments was measured at times of 5, 30, 60, and 120 min of reaction. The method established by Klamerth [40] was followed to carry out the quantitative analysis of residual H_2_O_2_, which is based on adding titanium oxysulfate to the samples, which causes a yellow color that varies in intensity in relation to the H_2_O_2_ concentration. The solutions were measured at a wavelength of 410 nm. The calibration curve was developed using H_2_O_2_ solutions with concentrations ranging from 0 to 3 mM/L.

The TOC of samples taken at different experimentation times was measured by direct injection of samples filtered with 0.2 mm syringe-driven filters into a Shimadzu 5000A TOC analyzer (Shimadzu Scientific Instruments Inc., Columbia, MD, USA).

### 3.7. MTX Formation By-Products

Samples at 0 and 120 min were analyzed by HPLC-MS to determine the chemical structure of possible MTX degradation byproducts. For comparison purposes, different equipment was used: (a) an Agilent 1100 HPLC with a UV detector coupled to an Agilent Trap XCT mass spectrometer (Agilent Technologies, Inc., Santa Clara, CA, USA); and (b) an HPLC Surveyor MS with an LTQ spectrometer (Thermo Fisher Scientific, Inc., Waltham, MA, USA).

## 4. Conclusions

Methotrexate is a folic acid analog and has been continually discharged into the environment as it is not considered in common treatments employed by wastewater treatment plants. 

The photolytic degradation of MTX after two hours of UV radiation in the absence of H_2_O_2_ was less than 17% in the three magnitudes of pH evaluated. The MTX degradation by photolysis in the presence of 3 mM/L H_2_O_2_ showed the best results obtained at a pH of 3.5, reaching a total degradation of 82.64%.

Kinetic analysis showed kinetic constants of 0.028 min^−1^, 0.013 min^−1^, and 0.027 min^−1^ for the photolysis process when adding 3 mM of peroxide for experiments with initial pHs of 3.5, 7, and 9.5, respectively. Calculated kinetic constants for photolytic MTX degradation without H_2_O_2_ addition were always lower.

The photocatalysis experiments showed a similar behavior to the photolysis experiments; the addition of H_2_O_2_ (3 mM/L) greatly improved the efficiency of pollutant degradation. After 120 min of reaction, a maximum of 65.73% was reached at a pH of 3.5.

Remarkably, photocatalytic kinetic constants were lower than those calculated for the photolytic process, with values for experiments when H_2_O_2_ was added of 0.016 min^−1^, 0.005 min^−1^, and 0.008 min^−1^ for initial pH values of 3.5, 7, and 9.5, respectively.

The statistical analysis showed that all factors considered (process, initial pH, peroxide addition, and experimentation time) were significant for MXT degradation (*p* < 0.05).

The residual hydrogen peroxide for MTX degradation in the photolysis experiments was 57.5%, while in the photocatalysis processes the H_2_O_2_ consumption rose to 76%, so at the experimental conditions presented in this work and 120 min of UV radiation, it can be reduced to 2.3 mM/L for photocatalysis and 1.8 mM/L for photolysis.

## Figures and Tables

**Table 1:** Results of the ANOVA, considering the degradation of methotrexate as a response variable, and the factors process, pH, addition of peroxide, and time as independent variables.

Factor	Degrees of Freedom	Sum of Squares	Mean Squares	F-Value	Pr (>F)
Process	1	1998	1998	8.934	0.00343
pH	2	1627	1627	7.276	0.00106
Peroxide addition	1	25,549	25,549	114.24	2 × 10^−16^
Time	1	11,511	11,511	51.471	7.85 × 10^−11^
Residuals	114	25,496	224		

**Table 2:** Experimental parameters and level values for the design of experiments for MTX degradation.

Variable	Units	Levels
Process	-	UV-C photolysis, UV-C photocatalysis
Initial pH	-	3.5, 7, 9.5
H_2_O_2_ addition	mM/L	0, 3
Time	min	0, 5, 10, 15, 20, 30, 45, 60, 90, 120

## Data Availability

Data are contained within the article.
